# Supplementary material for: Combining 3D single molecule localization strategies for reproducible bioimaging
Source: Nat Commun. 2019 Apr 30;10:1980. doi: 10.1038/s41467-019-09901-8 (PMC6491430; doi:10.1038/s41467-019-09901-8)
Supplement: Supplementary file 1 — Supplementary Information [file 41467_2019_9901_MOESM1_ESM.pdf]

Supplementary information

Combining 3D single molecule localization strategies for reproducible bioimaging

Cabriel et al.

Supplementary Figure 1: Influence of the depth and the astigmatism amplitude on the axial and lateral Cramér-Rao Lower Bound (CRLB) theoretical limits.

Supplementary Figure 2: Visualization of the hollowness of microtubules to measure the localization precision.

Supplementary Figure 3: Comparison of the lateral and axial CRLB for DONALD, weak astigmatism and DAISY.

Supplementary Figure 4: Long-term tracking of the axial drift.

Supplementary Figure 5: Influence of the remaining field aberrations on the axial detection after tilt correction.

Supplementary Figure 6: Measurement of the residual axial registration error after the correction.

Supplementary Figure 7: Measurement of the residual lateral registration error after the correction.

Supplementary Figure 8: Comparison of the 3D performances of standard astigmatism, DONALD and DAISY on the same sample.

Supplementary Figure 9: Lateral and axial histograms plotted on the clathrin spheres presented in **Fig. 4g–h**.

Supplementary Note 1: Influence of the molecule density per frame on the localization computation.

Supplementary Note 2: Fisher information and Cramér-Rao Lower Bounds.

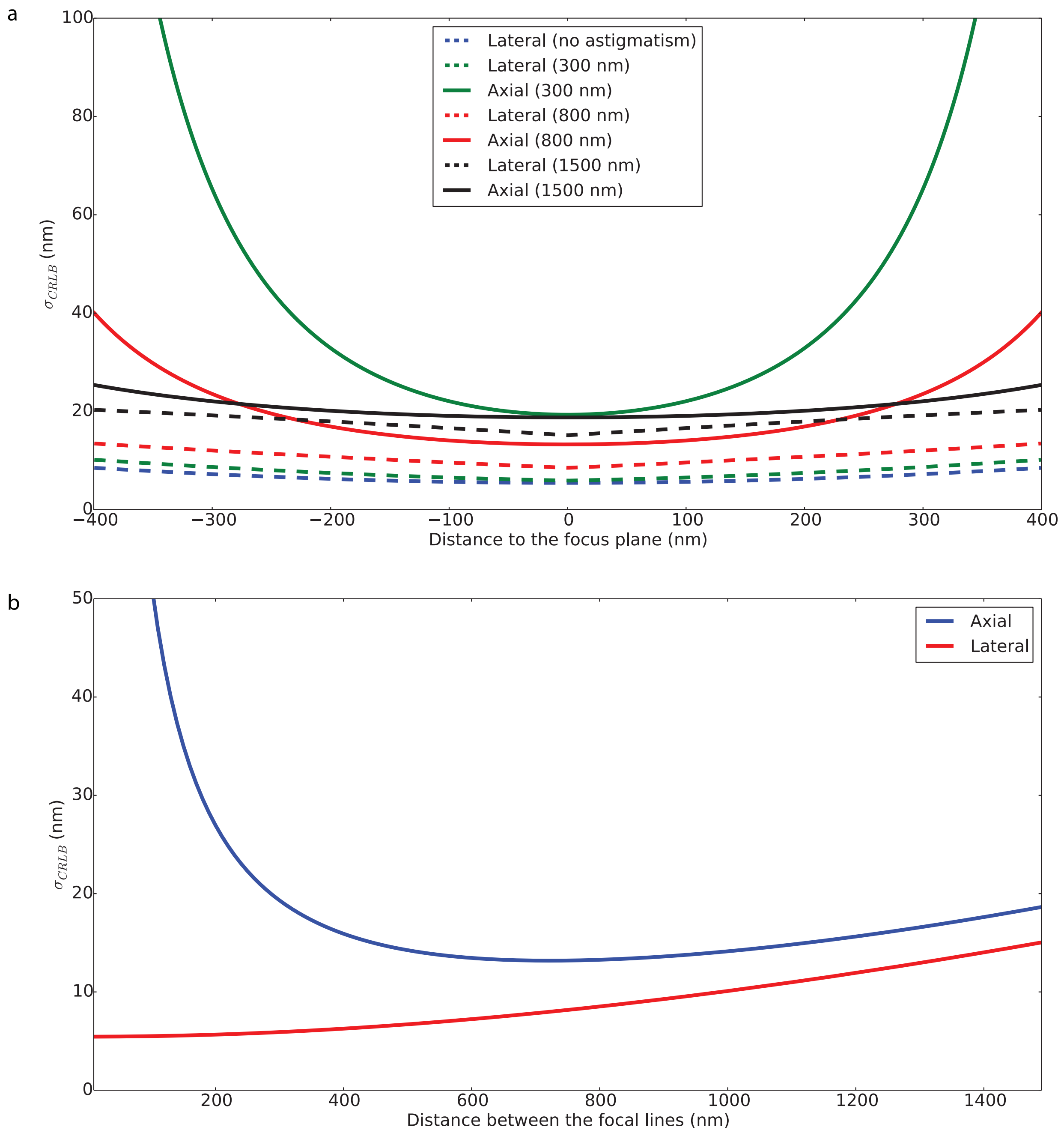

Supplementary Figure 1: Influence of the depth and the astigmatism amplitude on the axial and lateral Cramér-Rao Lower Bound (CRLB) theoretical limits for 2750 photons per PSF. **(a)** Variation of the localization precision with the distance to the focal plane for different astigmatism amplitudes (expressed as the distance between the two focal lines in the object space, 300 nm being a typical value found in the literature and 800 nm being the value used for DAISY). The solid and dashed lines stand for the axial and lateral precisions respectively. See **Supplementary Note** 2 for an explanation of the CRLB calculations. **(b)** Influence of the astigmatism amplitude on the best achievable axial and lateral precisions (i.e. CRLB values at $z = 0$). Note that the axial precision displays a minimum around 800 nm, which we chose as the astigmatism amplitude for DAISY to optimize the axial detection.

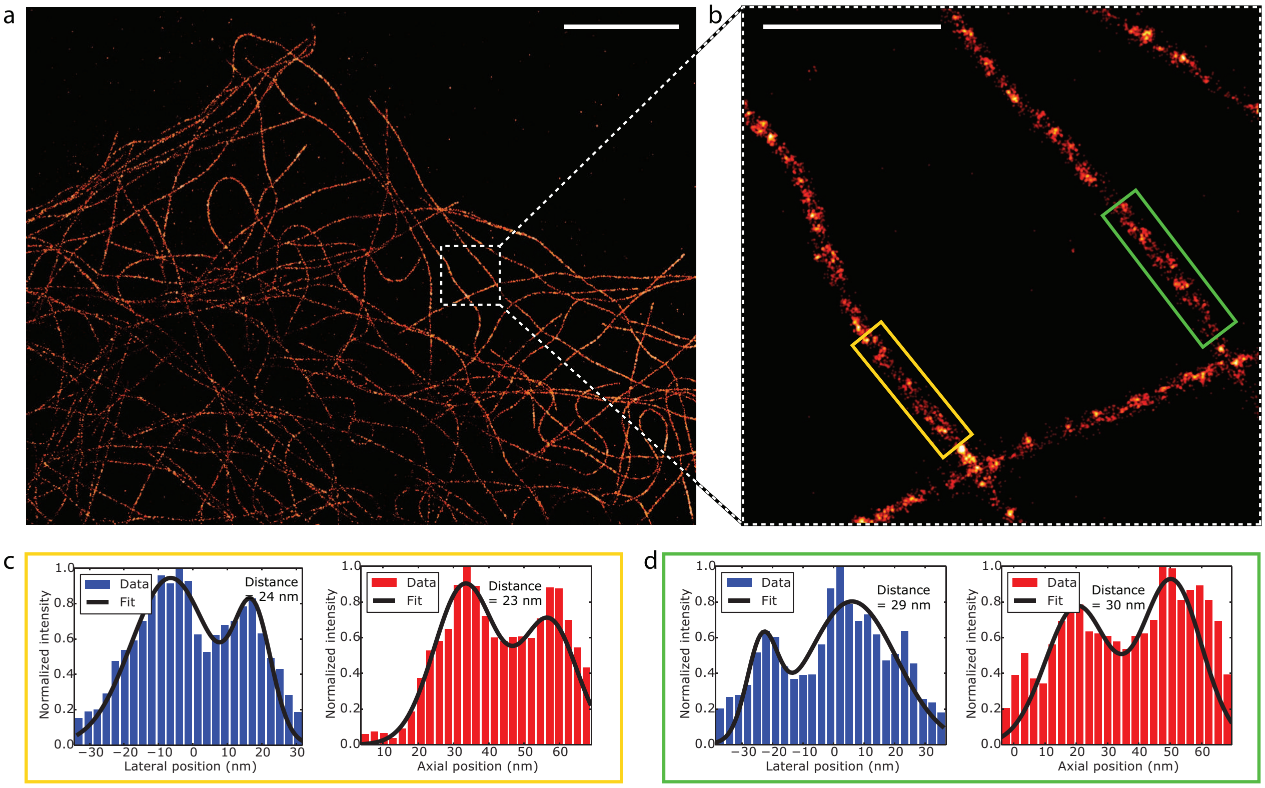

Supplementary Figure 2: Visualization of the hollowness of microtubules to measure the localization precision. **(a)** 2D super-localized image of COS-7 cells with α-tubulin labelled with AF647. The acquisition is the same as that presented in **Fig. 2i**. **(b)** Zoom on the boxed region displayed in **(a)**. The lateral (blue) and axial (red) histograms are then plotted in the yellow boxed region **(c)** and in the green boxed region **(d)**. Both the experimental data and the fitted profile with a double Gaussian function are displayed, as well as the distance between the two Gaussian peaks. The hollowness is clearly visible, and the distance between the peaks corresponds to a localization precision around 14–16 nm (see Supplementary Reference [1]: Supplementary Figure 7). Scale bars: 5 µm **(a)**, 1 µm **(b)**.

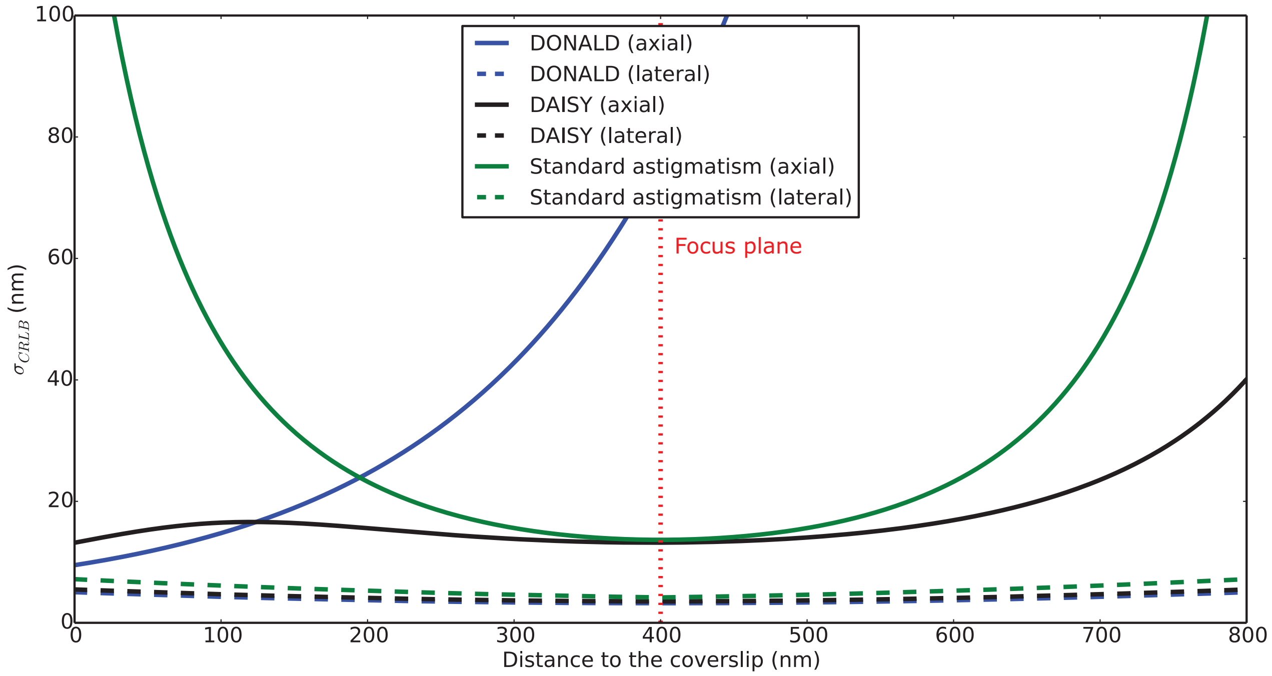

Supplementary Figure 3: Comparison of the lateral and axial CRLB for DONALD, standard astigmatism and DAISY as a function of the depth. The number of photons is 2750 for the UAF PSFs and 2750–5100 (depending on the axial position) for the EPI PSFs (similar to AF647), and the standard astigmatism corresponds to a 300-nm spacing between the two focal lines. The focus position is assumed to be 400 nm above the coverslip (typical experimental value), which is represented by the red dotted line. The solid and dashed lines stand for the axial and lateral precisions respectively. See **Supplementary Note** 2 for an explanation of the CRLB calculations.

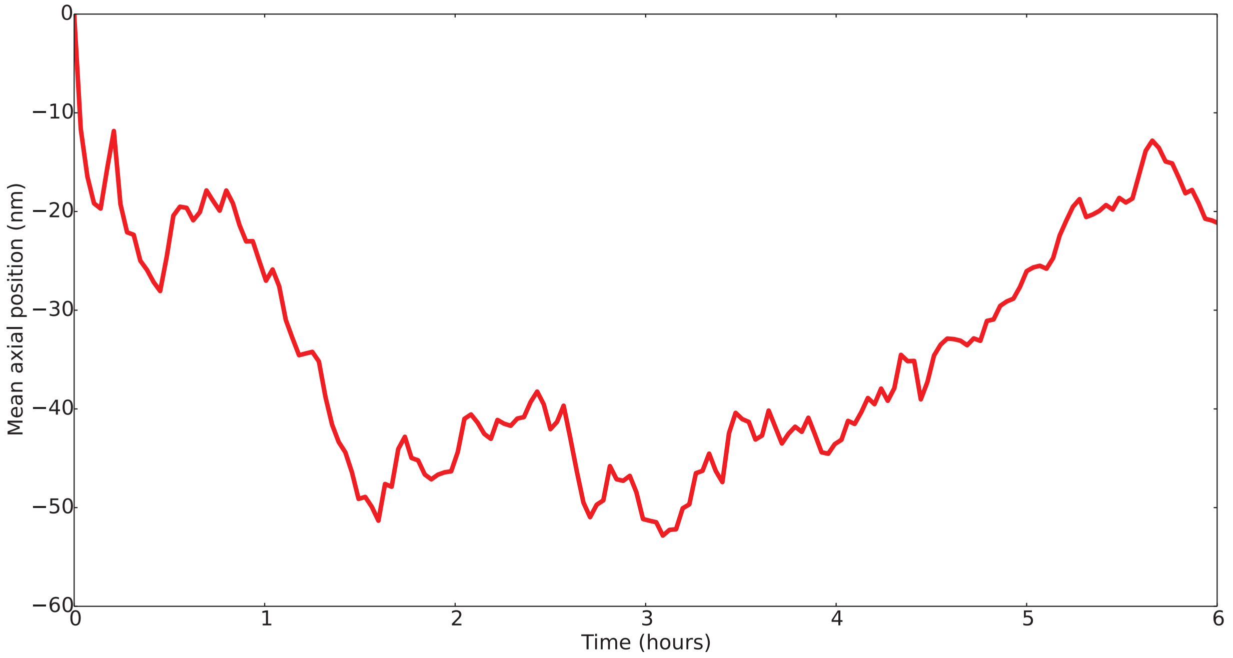

Supplementary Figure 4: Long-term tracking of the axial drift. The mean axial position of 100 nm diameter tetraspeck fluorescent beads (Thermo Fisher, T7279) over the imaged field is plotted as a function of time over approximately six hours. The results were averaged over 50 frames (i.e. 2.5 seconds) to suppress the influence of the localization uncertainty.

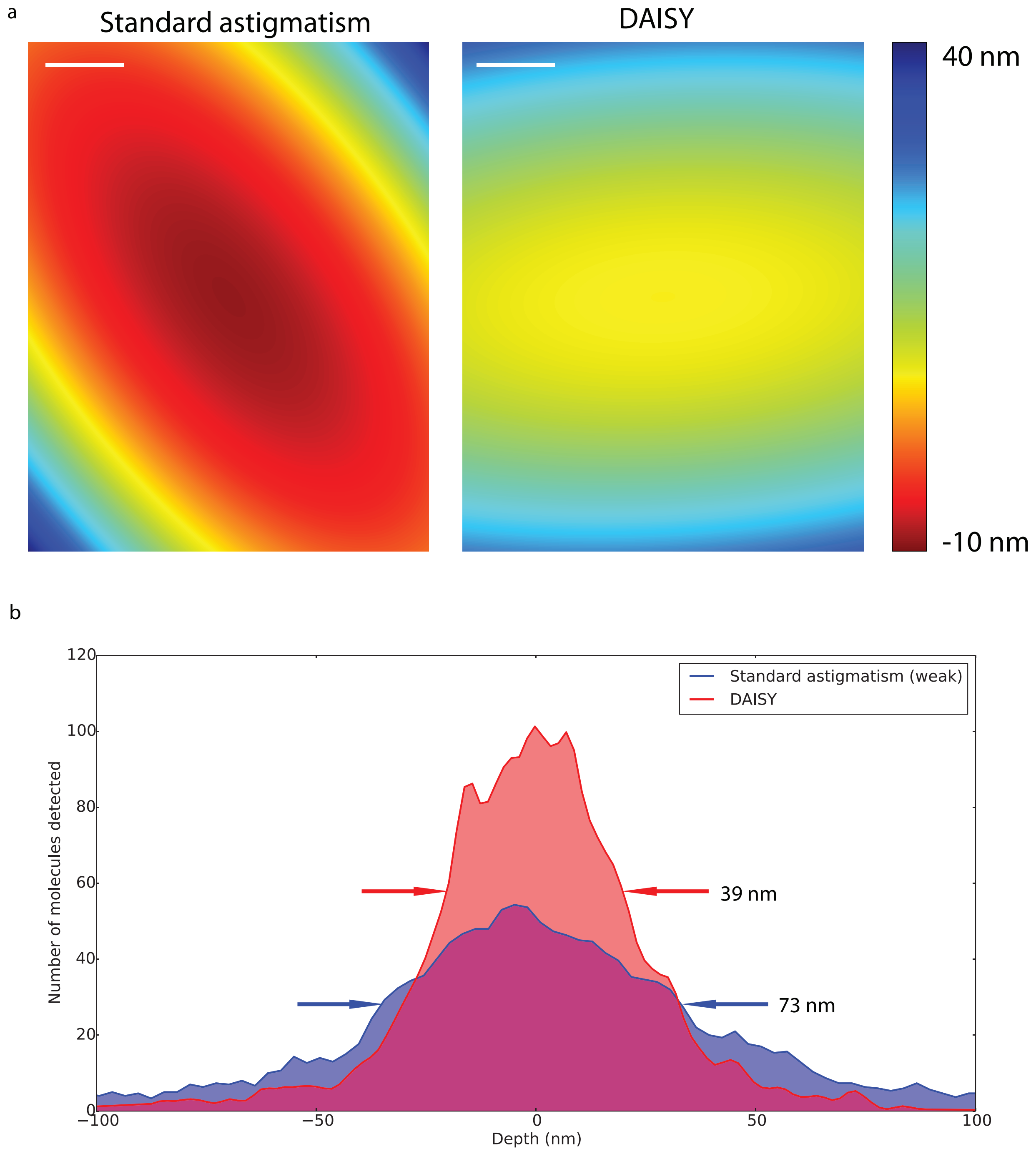

Supplementary Figure 5: Influence of the remaining field aberrations on the axial detection after tilt correction. **(a)** Interpolated depth maps of the axial positions measured for a sample of 20 nm dark red fluorescent beads deposited on a coverslip and averaged over 500 frames to suppress the influence of the localization precision. The results are plotted for both a typical astigmatism-based imaging (300 nm spacing between the two focal lines, close to the values encountered in the literature) and for DAISY. **(b)** The depth histograms are plotted over the 25-μm wide field for both the typical astigmatic detection (300 nm between the two focal lines) and for DAISY. The displayed widths stand for the full widths at half maximum. Scale bars: 5 μm.

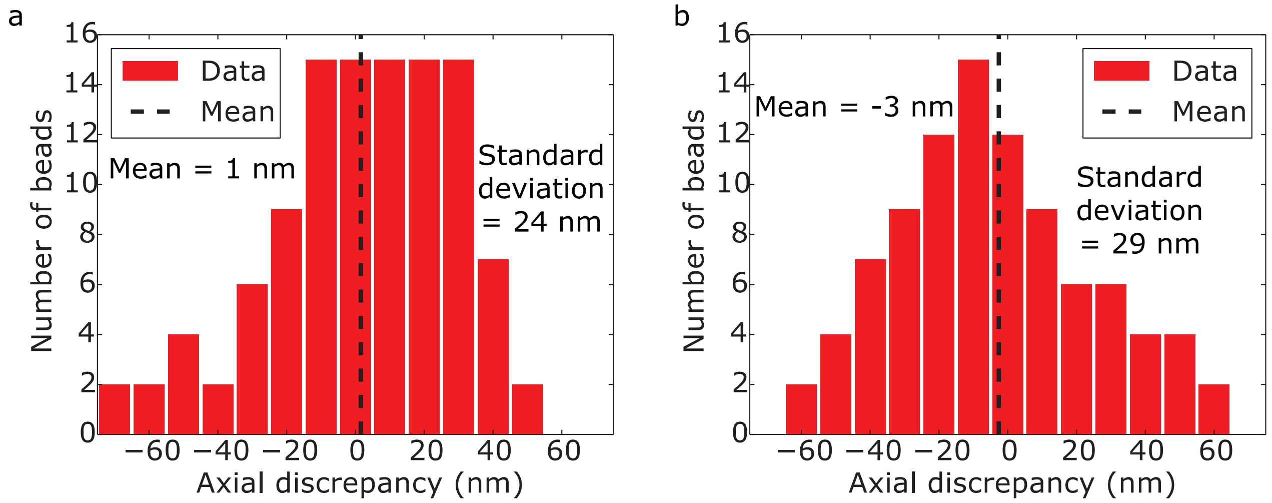

Supplementary Figure 6: Measurement of the residual axial registration error after the correction. **(a)** Histogram of the residual axial discrepancy (i.e. $z^{\text{astigmatic}} - z^{\text{SAF}}$) obtained with 40-nm diameter dark red fluorescent beads deposited at the coverslip. **(b)** Histogram of the residual axial discrepancy (i.e. $z^{\text{astigmatic}} - z^{\text{SAF}}$) obtained with 40-nm diameter dark red fluorescent beads randomly distributed in the volume and imaged over a 500-nm range above the coverslip capture range. In both cases, the axial positions were averaged over 500 frames to mitigate the influence of the localization precision.

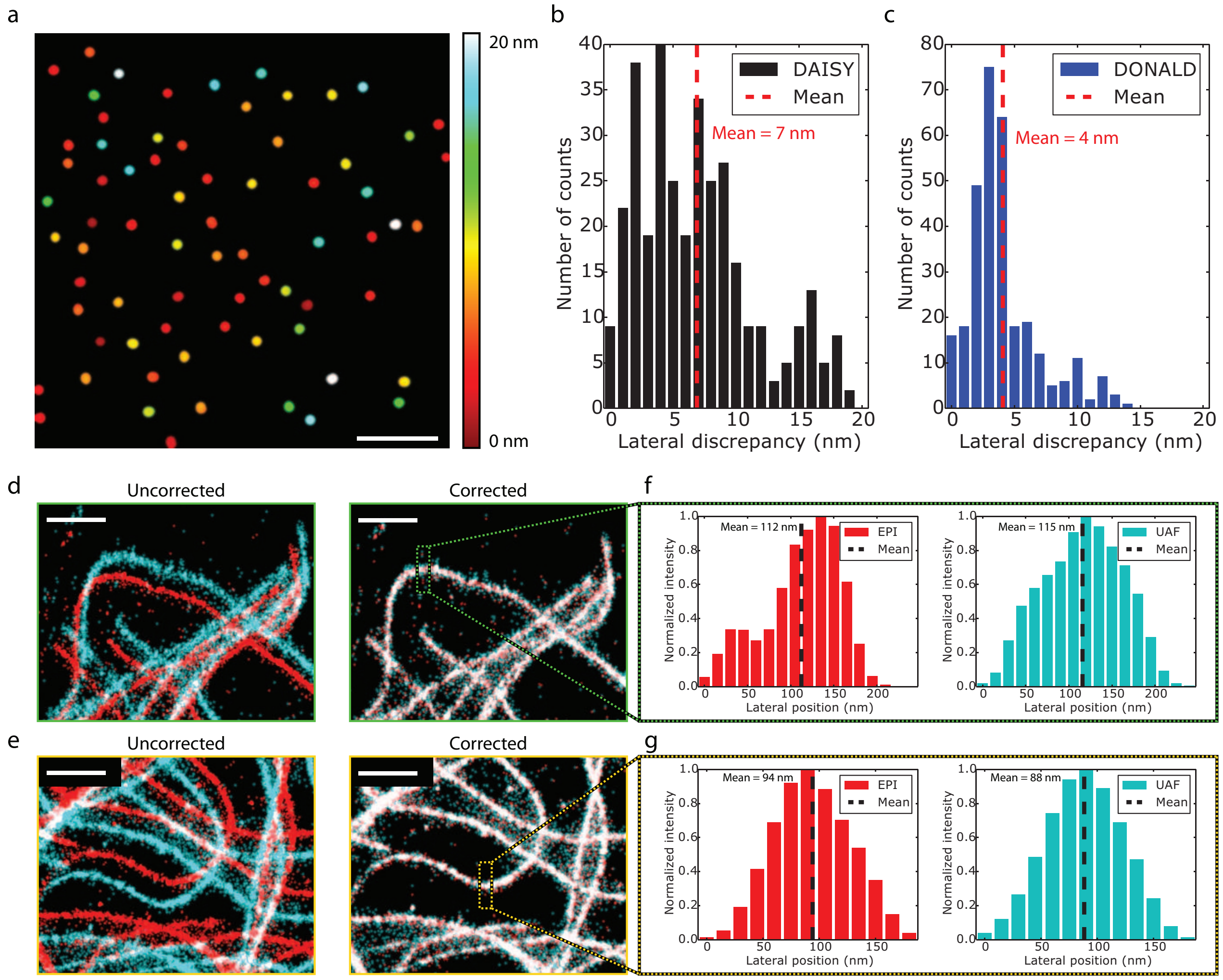

Supplementary Figure 7: Measurement of the residual lateral registration error after the correction. **(a–c)** Measurement on 40-nm diameter dark red fluorescent beads deposited at the coverslip (three acquisitions on different fields were stacked): **(a)** Map of the residual lateral error, **(b)** Residual lateral error histogram obtained with DAISY. As a comparison, the same measurement is provided for a DONALD acquisition (i.e. the same dual-view setup without the cylindrical lens) in **(c)**. In both cases, the axial positions were averaged over 500 frames to mitigate the influence of the localization precision. The residual discrepancies are slightly superior for DAISY (7 nm) than for DONALD (4 nm). We attribute this difference to either a PSF shape-dependent lateral bias (which would be larger for aberrated PSFs), or a residual influence of the localization uncertainty. **(d–g)** Measurements performed on the two microtubules regions of interest presented in **Fig. 2i**: **(d–e)** Superimposed 2D maps (red: EPI path, cyan: UAF path) before and after running the correction algorithm. The initial images display large discrepancies (around 500 nm) due to the magnification difference between the x and y axes, unlike the corrected. **(f–g)** EPI and UAF profiles along the axes displayed in **(d–e)** after correction. The residual discrepancy is below 6 nm. Scale bars: 5 µm **(a)**, 1 µm **(b–c)**.

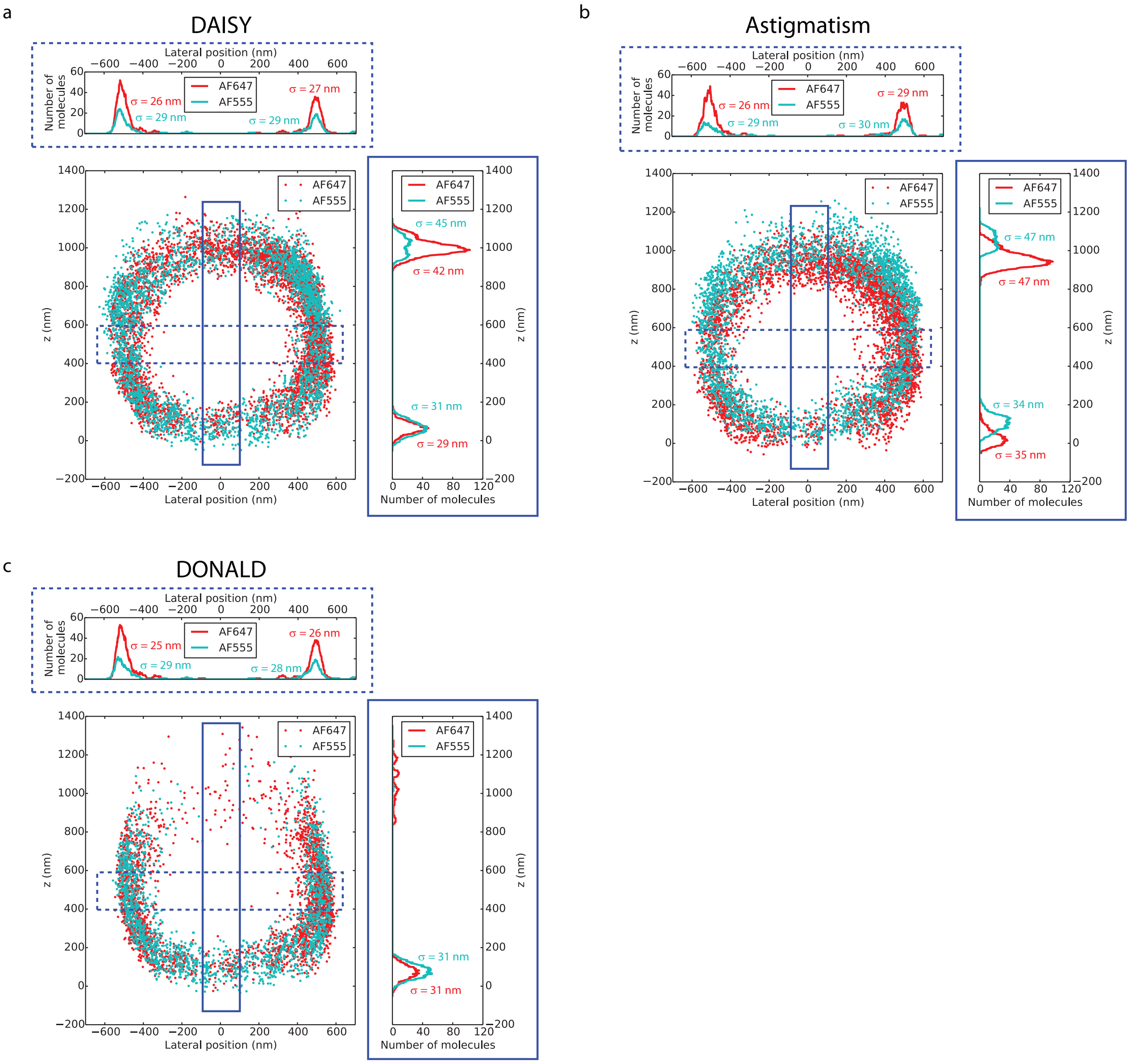

Supplementary Figure 8: Comparison of the 3D performances of **(a)** DAISY, **(b)** uncorrected astigmatism and **(c)** DONALD on a sample of living *E. coli* bacteria labeled with AF647 and AF555 at the membrane (see **Fig. 3a–c** and **Methods**). The x-z slices along the line displayed in **Fig. 3a** and the axial and lateral profiles in the boxed regions are plotted. The σ values stand for the standard deviation of the distributions. Like DAISY, DONALD features an absolute detection, unsensitive to both chromatic aberration and axial drift. However, the axial precision deteriorates sharply with the depth due to the decay of the SAF signal; thus the top half of the sample (beyond 500 nm) is hardly visible. Uncorrected astigmatism has the same capture range as DAISY, but since it lacks the absolute information, it exhibits an axial shift between the two colors as well as a broadening of the histogram widths due to the axial drift.

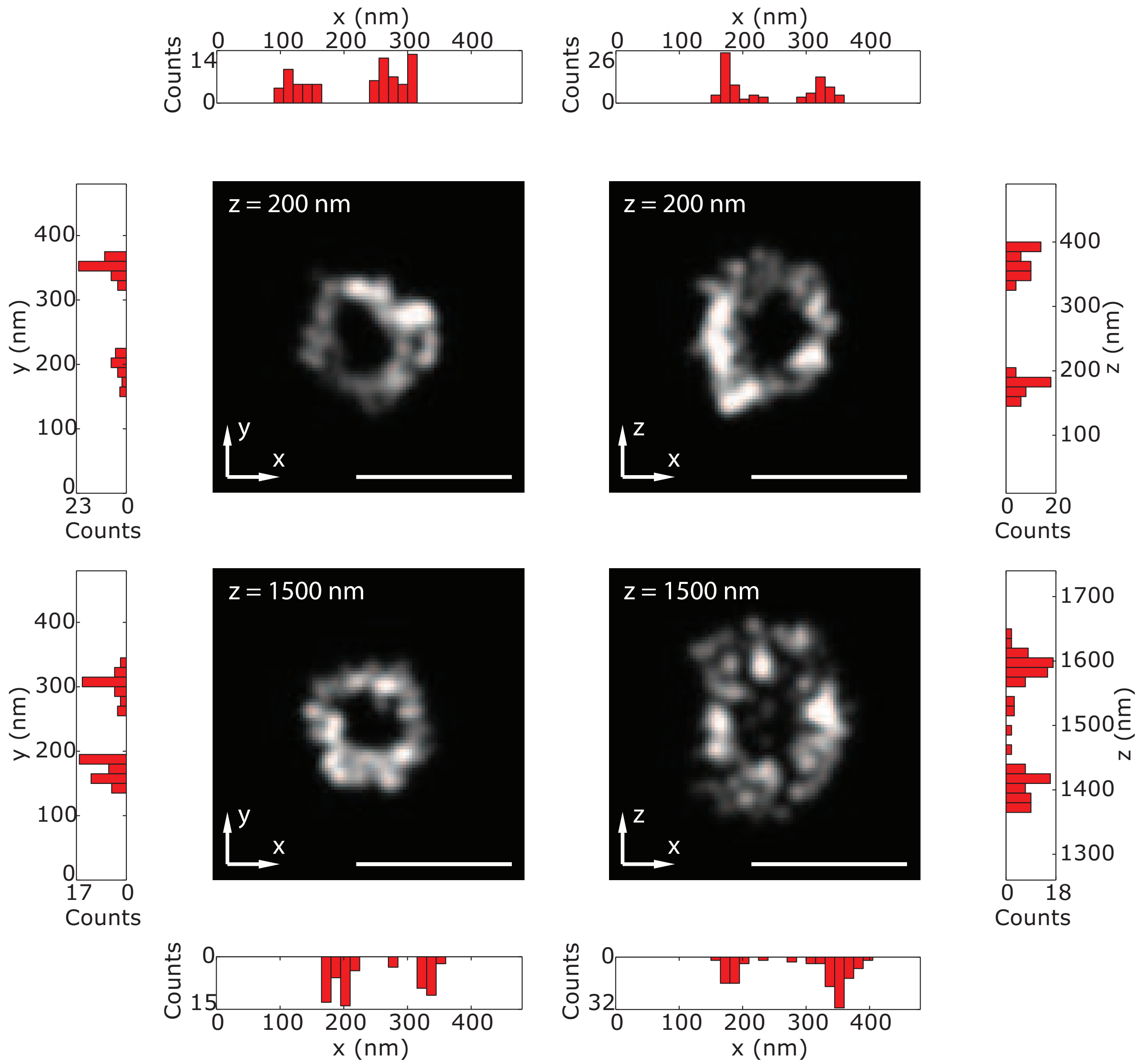

Supplementary Figure 9: Lateral and axial histograms plotted on the clathrin spheres presented in **Fig. 4g–h**. The histograms are plotted along lines at the center of the spheres to highlight their hollowness. Scale bars: 250 nm.

Supplementary Note 1: Influence of the molecule density per frame on the localization computation

To assess the impact of the molecule density per frame on the localization performance of our algorithm and filtering, we simulated DAISY PSFs on a 30 µm x 30 µm field with 100 nm pixels. The PSFs were simulated as elliptical gaussians for the UAF channel and circular gaussians for the EPI channel with realistic PSF sizes matching those of experimental data obtained with AF647. All the sources were considered at the coverslip ($z = 0$ nm) and all the PSFs had the same intensity (2750 UAF photons, 2750–5100 EPI photons depending on the axial position of the source). In order to decouple the effect of the molecule density from the localization precision, we did not add background or Poisson noise. The lateral positions were uniformly distributed over the field. The number of PSFs per frame ranged from 1 (1.1 10^{-3} molecules.µm^{-2}) to 1000 (1.1 molecules.µm^{-2}). The generated positions were recorded for later use.
Then the localization was run on the generated data and the filters (size and anisotropy of the unastigmatic EPI PSFs, distance between PSF neighboring pairs) were applied and the number of remaining detections was compared to the number of generated molecules to calculate the fraction of missed/discarded localizations (**Supplementary Fig. 10a**). Among the remaining localizations, those displaying a 3D distance to the expected position superior to 50 nm were flagged as wrong detections and their fraction among all the localizations after filtering was displayed in **Supplementary Fig. 10a** in the cases of the SAF and astigmatic detections. Finally, the lateral and axial (for the SAF and the astigmatic detections) median distances to the expected positions were displayed in **Supplementary Fig. 10b**.

As expected, the numbers of missed/discarded localizations and wrong detections increase with the density, but the latter remains quite low for reasonable densities (under 15 % below 0.3 molecules.µm^{-2}). Similarly, the lateral and axial position discrepancies increase with the density. Realistic dSTORM conditions correspond to densities around 10^{-2}–10^{-1} molecules.µm^{-2}. At such densities, the number of missed/rejected localizations can account for up to 40 % of the total number of localizations, but the number of wrong detections remains minimal (below 4 %). Besides, the errors on the measured positions are rather low (inferior to 4 nm in the lateral and 1 nm in the axial direction, both in SAF and astigmatism).
These results can prove useful to optimize the acquisition conditions—especially the composition of the imaging buffer in dSTORM, the concentration of imager strands in DNA-PAINT or the activation power in PALM, as well as the exposure time for all these methods. In DNA-PAINT acquisitions, relatively high molecule densities (up to 0.3 molecules.µm^{-2}) can be used to speed up acquisitions, as long as the localization error remains below the localization precision. On the contrary, in PALM experiments, the number of photoactivable molecules is often low and in order to minimize the number of missed/discarded molecules, the molecule density should be kept low (inferior to 3 10^{-2} molecules.µm^{-2}). Depending on the sensitivity of the fluorophores used to photobleaching, dSTORM acquisitions can match either of the two previously described cases.

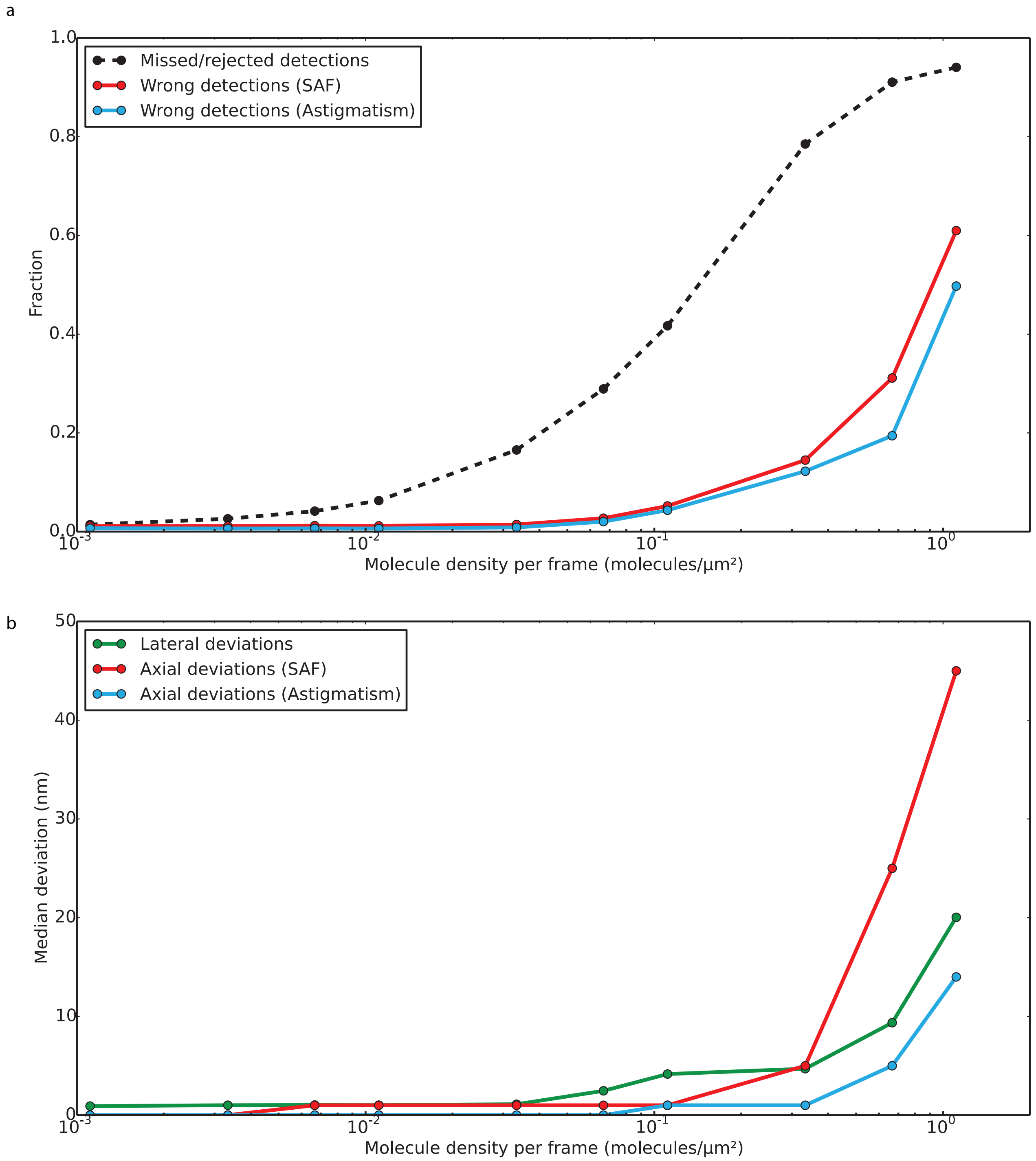

Supplementary Figure 10: Influence of the molecule density per frame on the localization computation. **(a)** Fraction of localizations missed or discarded (black dashed line) and fraction of wrong detections (blue and red solid lines) as a function of the molecule density on each frame. **(b)** Median lateral and axial discrepancies between the real and the measured positions as a function of the molecule density on each frame.

Supplementary Note 2: Fisher information and Cramér-Rao Lower Bounds

To determine the theoretical limits of our method, we calculated the Fisher information and the Cramér-Rao Lower Bounds (CRLB) of both the SAF and astigmatic axial detections, as well as that of the lateral detection in order to have access to the theoretical limits of DAISY.

1 Fisher information and CRLB for SAF

We used the same approach as Balzarotti *et al.*[2] to calculate the Fisher information for the supercritical angle fluorescence signal and the associated Cramér-Rao Lower Bounds. Considering an emitter at the position $\mathbf{r}$ exposed to K different illuminations, each photon acquired n_i $(i \in [0, K-1])$ follows a Poissonian statistics with a mean λ_i that depends on the illumination. The authors demonstrated that the components of the parameter vector with negligible dark count can be expressed as:

$$p_i^{(0)}(\mathbf{r}) = \frac{\lambda_i}{\sum_{j=0}^{K-1} \lambda_j} \text{ with } (i \in [0, K-1]) \tag{1}$$

Adding the background signal, this becomes:

$$p_i(\mathbf{r}) = \frac{\lambda_i + \lambda_{bi}}{\sum_{j=0}^{K-1} \left(\lambda_j + \lambda_{bj}\right)} \tag{2}$$

This equation can be simplified:

$$\begin{aligned} p_i(\mathbf{r}) &= \frac{\text{SBR}(\mathbf{r})}{\text{SBR}(\mathbf{r})+1} \frac{\lambda_i}{\sum_{j=0}^{K-1} \lambda_j} + \frac{1}{\text{SBR}(\mathbf{r})+1} \frac{\lambda_{bi}}{\sum_{j=0}^{K-1} \lambda_{bj}} \\ &= \frac{\text{SBR}(\mathbf{r})}{\text{SBR}(\mathbf{r})+1} p_i^{(0)} + \frac{1}{\text{SBR}(\mathbf{r})+1} \frac{1}{K} \end{aligned} \tag{3}$$

where $\text{SBR}(\mathbf{r}) = \frac{\sum_{j=0}^{K-1} \lambda_j}{\sum_{j=0}^{K-1} \lambda_{bj}}$ represents the signal to background ratio. Balzarotti *et al.* showed that the Fisher matrix can be expressed in a simple form:

$$F_{\mathbf{r}} = N \sum_{i=0}^{K-1} \frac{1}{p_i} \begin{bmatrix} \left(\frac{\partial p_i}{\partial \mathbf{r}_1}\right)^2 & \cdots & \frac{\partial p_i}{\partial \mathbf{r}_1} \frac{\partial p_i}{\partial \mathbf{r}_d} \\ \vdots & \ddots & \vdots \\ \frac{\partial p_i}{\partial \mathbf{r}_d} \frac{\partial p_i}{\partial \mathbf{r}_1} & \cdots & \left(\frac{\partial p_i}{\partial \mathbf{r}_d}\right)^2 \end{bmatrix} \tag{4}$$

The Fisher information matrix gives access to a lower bound for the covariance matrix $\Sigma(\mathbf{r})$. The arithmetic mean of the eigenvalues $\widetilde{\sigma}_{\text{CRLB}}$ of the lower bound matrix is interpreted as a performance metric:

$$\Sigma(\mathbf{r}) \geq \Sigma_{\text{CRLB}}(\mathbf{r}) = F_{\mathbf{r}}^{-1} \tag{5a}$$

$$\widetilde{\sigma}_{\text{CRLB}} = \sqrt{\frac{1}{d} \text{tr}(\Sigma_{\text{CRLB}}(\mathbf{r}))} \tag{5b}$$

where d is the number of dimensions considered and N is the total acquired photon number. These results can be transposed to our model provided a few modifications. Rather than considering different illuminations, we consider a sampling of the signal in two parts: one (EPI signal, noted $i = 0$) dependent on the z position of the emitter and one (UAF signal, noted $i = 1$) independent of z. In this case, the Fisher matrix takes the form of a scalar:

$$F_z = N \left(\frac{1}{p_0} \left(\frac{\partial p_0}{\partial z} \right)^2 + \frac{1}{p_1} \left(\frac{\partial p_1}{\partial z} \right)^2 \right) \tag{6}$$

$p_i(z)$ is provided by **Supplementary equation** (3):

$$p_i(z) = \frac{\mathrm{SBR}(z)}{\mathrm{SBR}(z)+1} p_i^{(0)} + \frac{0.5}{\mathrm{SBR}(z)+1} \tag{7}$$

Supplementary equation (3) can be differentiated:

$$\frac{\partial p_i(z)}{\partial z} = \frac{\partial \mathrm{SBR}(z)}{\partial z} \frac{p_i^{(0)} - 0.5}{(\mathrm{SBR}(z)+1)^2} + \frac{\partial p_i^{(0)}}{\partial z} \frac{\mathrm{SBR}(z)}{\mathrm{SBR}(z)+1} \tag{8}$$

First, we use the theoretical dependence of the SAF signal versus the z position by performing simulations based on the work of Wai Teng Tang *et al.* [3]. By fitting the simulation results, we assume that the ratio between the SAF and UAF photon numbers can be approximated as follows for a numerical aperture of 1.49 and an fluorescence wavelength λ_{fluo}:

$$\frac{N_{\text{SAF}}}{N_{\text{UAF}}} = 0.85 \ \exp\left(-\frac{z}{0.24 \ \lambda_{\text{fluo}}} \right) \tag{9}$$

The signal of an emitter is divided in two parts so as to separate the UAF from the EPI fluorescence. In this case, the mean of the Poisson distribution for each part can be expressed as:

$$\begin{aligned} \lambda_{\text{EPI}} = \lambda_0 &= \frac{(N_{\text{UAF}} + N_{\text{SAF}})}{2} \\ &= \frac{N_{\text{UAF}}}{2} \left(1 + 0.85 \exp(-\alpha z)\right) \end{aligned} \tag{10a}$$

$$\lambda_{\text{UAF}} = \lambda_1 = \frac{N_{\text{UAF}}}{2} \tag{10b}$$

with $\alpha = \frac{1}{0.24 \ \lambda_{\text{fluo}}}$. Thses terms can be used in **Supplementary equation** (1) to obtain the two components of the parameter vector with neglected Gaussian noise:

$$p_0^{(0)}(z) = \frac{1 + 0.85 \exp(-\alpha z)}{2 + 0.85 \exp(-\alpha z)} \tag{11a}$$

$$p_1^{(0)}(z) = \frac{1}{2 + 0.85 \exp(-\alpha z)} \tag{11b}$$

At this point, a background noise term B has to be introduced in the calculation. B is a photon number associated to an optical signal produced mainly by fluorescent probes located outside the focal plane and is approximated to 200 photons per channel in our calculations. B represents $\lambda_{bj} = \lambda_b$, considered constant for each channel. We define the SBR(z) as :

$$\mathrm{SBR}(z) = \frac{\sum_{j=0}^{1} \lambda_j}{\sum_{j=0}^{1} \lambda_{bj}} = \frac{N_{\mathrm{UAF}}\,(2 + 0.85\exp(-\alpha z))}{4B} \tag{12}$$

Finally, we can extract the expression of the Fisher information and the CRLB:

$$F = N\left(\frac{1}{\frac{\mathrm{SBR}(z)}{\mathrm{SBR}(z)+1}p_0^{(0)} + \frac{0.5}{\mathrm{SBR}(z)+1}}\left(\frac{\partial \mathrm{SBR}(z)}{\partial z}\frac{p_0^{(0)} - 0.5}{(\mathrm{SBR}(z)+1)^2} + \frac{\partial p_0^{(0)}}{\partial z}\frac{\mathrm{SBR}(z)}{\mathrm{SBR}(z)+1}\right)^2 + \frac{1}{\frac{\mathrm{SBR}(z)}{\mathrm{SBR}(z)+1}p_1^{(0)} + \frac{0.5}{\mathrm{SBR}(z)+1}}\left(\frac{\partial \mathrm{SBR}(z)}{\partial z}\frac{p_1^{(0)} - 0.5}{(\mathrm{SBR}(z)+1)^2} + \frac{\partial p_1^{(0)}}{\partial z}\frac{\mathrm{SBR}(z)}{\mathrm{SBR}(z)+1}\right)^2\right) \tag{13a}$$

$$\Delta z_{\mathrm{CRLB}}^{\mathrm{SAF}} = \sqrt{\frac{1}{F(z)}} \tag{13b}$$

with the different parameters:

$$\frac{\partial p_0^{(0)}}{\partial z} = -\frac{0.85\,\alpha\,\exp(-\alpha z)}{(2 + 0.85\exp(-\alpha z))^2} \tag{14a}$$

$$\frac{\partial p_1^{(0)}}{\partial z} = \frac{0.85\,\alpha\,\exp(-\alpha z)}{(2 + 0.85\exp(-\alpha z))^2} \tag{14b}$$

$$\frac{\partial \mathrm{SBR}}{\partial z} = -\frac{N_{\mathrm{UAF}}\,0.85\,\alpha\,\exp(-\alpha z)}{4B} \tag{14c}$$

2 CRLB for astigmatism

The Cramér-Rao Lower Bound for the astigmatic detection is directly computed from the work of Rieger and Stallinga [4]. We consider that an astigmatic PSF can be approximated by an elliptical Gaussian PSF with different widths in x and y, noted w_x and w_y:

$$H = \frac{N}{2\pi w_x w_y}\exp -\left(\frac{(x-x_0)^2}{2w_x^2} + \frac{(y-y_0)^2}{2w_y^2}\right) \tag{15}$$

From **Supplementary equation** (15), the CRLB for the $w_{x,y}$ parameters can be approximated with the semi-exact formula:

$$\left(\Delta w_{x,y}\right)^2 \approx \frac{w_{x,y}^2}{2N}\left(1 + 8\tau + \sqrt{\frac{9\tau}{1+4\tau}}\right) \tag{16}$$

where τ is approximately equal to the ratio between the peak and background intensities (a being pixel size):

$$\tau = \frac{2\pi b(w_x w_y + a^2/12)}{Na^2} \tag{17}$$

The authors derive the axial detection position from the focus S curve:

$$f = \frac{w_x^2 - w_y^2}{w_x^2 + w_y^2} = \frac{2lz}{l^2 + d^2 + z^2} \tag{18}$$

where d stands for the focal depth and $2l$ is the distance between the focal lines. Usually, these two parameters are obtained by experimental measurements. The CRLB for the axial position is expressed as follows:

$$\left(\Delta z^{\text{astigmatic}}\right)^2 = \frac{\left(l^2 + d^2 + z^2\right)^4}{4l^2\left(l^2 + d^2 - z^2\right)^2}\left(\Delta f\right)^2 \tag{19a}$$

$$\left(\Delta f\right)^2 = \left(1 - f^2\right)\left(\left(\frac{\Delta w_x}{w_x}\right)^2 + \left(\frac{\Delta w_y}{w_y}\right)^2\right) \tag{19b}$$

By combining **Supplementary equations** (16), (18), (19a) and (19b), the final expression of the CRLB for the axial position of astigmatic method reads:

$$\left(\Delta z^{\text{astigmatic}}\right)^2 = \frac{1}{N}\frac{\left(l^2 + d^2 + z^2\right)^4}{4l^2\left(l^2 + d^2 - z^2\right)^2}\left(1 - \left(\frac{2lz}{l^2 + d^2 + z^2}\right)^2\right)\left(1 + 8\tau + \sqrt{\frac{9\tau}{1 + 4\tau}}\right) \tag{20}$$

3 CRLB for DAISY

In DAISY, the axial positions from SAF and astigmatism are merged according their uncertainties in order to optimize the final precision (see **Methods**, **Position merging** section, **equation** (3)):

$$\begin{aligned} z^{\text{DAISY}} &= \left(\frac{z^{\text{SAF}}}{\left(\Delta z^{\text{SAF}}\right)^2} + \frac{z^{\text{astigmatic}}}{\left(\Delta z^{\text{astigmatic}}\right)^2}\right) \Big/ \left(\frac{1}{\left(\Delta z^{\text{SAF}}\right)^2} + \frac{1}{\left(\Delta z^{\text{astigmatic}}\right)^2}\right) \\ &= \pi^{\text{SAF}} z^{\text{SAF}} + \pi^{\text{astigmatic}} z^{\text{astigmatic}} \end{aligned} \tag{21}$$

where π^{SAF} and $\pi^{\text{astigmatic}}$ are the relative weights of the SAF and astigmatic information sources (note that these weights vary with the axial position):

$$
\begin{aligned}
\pi^{\text{SAF}} &= \frac{1}{(\Delta z^{\text{SAF}})^2} \Big/ \left(\frac{1}{(\Delta z^{\text{SAF}})^2} + \frac{1}{(\Delta z^{\text{astigmatic}})^2} \right) \\
\pi^{\text{astigmatic}} &= \frac{1}{(\Delta z^{\text{astigmatic}})^2} \Big/ \left(\frac{1}{(\Delta z^{\text{SAF}})^2} + \frac{1}{(\Delta z^{\text{astigmatic}})^2} \right)
\end{aligned}
\tag{22}
$$

The CRLB for DAISY then reads:

$$
\left(\Delta z^{\text{DAISY}}\right)^2 = \left(\pi^{\text{SAF}}\right)^2 \left(\Delta z^{\text{SAF}}\right)^2 + \left(\pi^{\text{astigmatic}}\right)^2 \left(\Delta z^{\text{astigmatic}}\right)^2 \tag{23}
$$

4 CRLB for the lateral detection

The lateral lower bound was obtained using the same assumptions (PSF shape, photon number, background) than those described for the axial detection. We used the formula provided in [5]:

$$
(\Delta x, y)^2 = \frac{w_{x,y}^2 + a^2/12}{N} \left(1 + 4\tau + \sqrt{\frac{2\tau}{1+4\tau}} \right) \tag{24}
$$

with τ defined as in **Supplementary equation** (17).
Like the axial position, the lateral position results from the merging of the measured lateral UAF and EPI positions (see **Methods**, **Position merging** section, **equations** (1) and (2)). Thus it can be written as a weighted sum:

$$
\begin{aligned}
x^{\text{DAISY}} &= \pi_x^{\text{UAF}} x^{\text{UAF}} + \pi_x^{\text{EPI}} x^{\text{EPI}} \\
y^{\text{DAISY}} &= \pi_y^{\text{UAF}} y^{\text{UAF}} + \pi_y^{\text{EPI}} y^{\text{EPI}}
\end{aligned}
\tag{25}
$$

where $\pi_{x,y}^{\text{UAF}}$ and $\pi_{x,y}^{\text{EPI}}$ are the relative weights of the UAF and EPI information sources for the x and y positions respectively (note that these weights vary with the axial position):

$$
\begin{aligned}
\pi_{x,y}^{\text{UAF}} &= \frac{1}{(\Delta(x,y)^{\text{UAF}})^2} \Big/ \left(\frac{1}{(\Delta(x,y)^{\text{UAF}})^2} + \frac{1}{(\Delta(x,y)^{\text{EPI}})^2} \right) \\
\pi_{x,y}^{\text{EPI}} &= \frac{1}{(\Delta(x,y)^{\text{EPI}})^2} \Big/ \left(\frac{1}{(\Delta(x,y)^{\text{UAF}})^2} + \frac{1}{(\Delta(x,y)^{\text{EPI}})^2} \right)
\end{aligned}
\tag{26}
$$

As a result, the CRLB finally reads:

$$
\begin{aligned}
\left(\Delta x^{\text{DAISY}}\right)^2 &= \left(\pi^{\text{UAF}}\right)^2 \left(\Delta x^{\text{UAF}}\right)^2 + \left(\pi^{\text{EPI}}\right)^2 \left(\Delta x^{\text{EPI}}\right)^2 \\
\left(\Delta y^{\text{DAISY}}\right)^2 &= \left(\pi^{\text{UAF}}\right)^2 \left(\Delta y^{\text{UAF}}\right)^2 + \left(\pi^{\text{EPI}}\right)^2 \left(\Delta y^{\text{EPI}}\right)^2
\end{aligned}
\tag{27}
$$

Supplementary references

[1] N. Bourg, C. Mayet, G. Dupuis, T. Barroca, P. Bon, S. Lécart, E. Fort, and S. Lévêque-Fort, "Direct optical nanoscopy with axially localized detection," *Nature Photonics*, vol. 9, pp. 587–593, aug 2015.

[2] F. Balzarotti, Y. Eilers, K. C. Gwosch, A. H. Gynnå, V. Westphal, F. D. Stefani, J. Elf, and S. W. Hell, "Nanometer resolution imaging and tracking of fluorescent molecules with minimal photon fluxes," *Science*, vol. 355, pp. 606–612, dec 2016.

[3] W. T. Tang, E. Chung, Y.-H. Kim, P. T. C. So, and C. J. R. Sheppard, "Investigation of the point spread function of surface plasmon-coupled emission microscopy," *Optics Express*, vol. 15, no. 8, p. 4634, 2007.

[4] B. Rieger and S. Stallinga, "The lateral and axial localization uncertainty in super-resolution light microscopy," *ChemPhysChem*, vol. 15, no. 4, pp. 664–670, 2014.

[5] S. Stallinga and B. Rieger, "The effect of background on localization uncertainty in single emitter imaging," in *2012 9th IEEE International Symposium on Biomedical Imaging (ISBI)*, pp. 988–991, IEEE, may 2012.
